# Perthes Disease in a Child With Osteogenesis Imperfecta From a Rare Genetic Variant: A Case Report

**DOI:** 10.3389/fgene.2022.920950

**Published:** 2022-07-08

**Authors:** Pan Hong, Xiaolong Zhao, Ruikang Liu, Saroj Rai, Yingying Song, Ruijing Xu, Jin Li

**Affiliations:** ^1^ Department of Orthopaedic Surgery, Union Hospital, Tongji Medical College, Huazhong University of Science and Technology, Wuhan, China; ^2^ Department of Orthopaedics, First Hospital of Wuhan, Wuhan, China; ^3^ Department of Endocrinology, Union Hospital, Tongji Medical College, Huazhong University of Science and Technology, Wuhan, China; ^4^ Department of Orthopaedics and Trauma Surgery, Karama Medical Center, Dubai, United Arab Emirates; ^5^ Medical Department of Hubei University of Science and Technology, Xianning, China

**Keywords:** osteogenesis imperfecta, Perthes disease, Legg-Calve-Perthes Disease, *COL1A1* gene, case report

## Abstract

**Background:** Although certain genetic components have been reported as contributing factors for Perthes disease, its etiology remains unclear. We present a rare case of Perthes disease in a child with osteogenesis imperfecta (OI) caused by a mutation in the *COL1A1* gene *(NM_000088):exon25:c.1726C>T, (p.Gln576X)*.

**Case presentations:** A 7-year-old boy was initially treated at our medical facility in March 2016 with a history of chronic pain in right hip joint and limping for a year. He was diagnosed as Perthes disease in the right hip joint. He underwent acetabular osteotomy and ipsilateral proximal femoral varus osteotomy for better containment. During the follow-ups, the right hip demonstrated a normal range of motion without pain, and the pelvic X-ray demonstrated Stulberg Type II hip joint with a round femoral head. In the latest admission in 2022, he suffered from a right femoral shaft fracture after petty violence. After reviewing his medical history, he was suspected of having OI. The whole exome sequencing demonstrated a gene mutation in *COL1A1* (OMIM 166200) and confirmed the diagnosis of OI. Telescopic nailing was used to treat the femoral shaft fracture. After the nailing of the right femur, the appearance of the lower extremity seemed normal and symmetrical.

**Conclusion:** This study revealed that there might be an association between OI and Perthes disease. Our case report enriches the phenotypes of osteogenesis imperfecta and provides insight into the pathogenesis of LCPD.

## Background

Osteogenesis imperfecta (OI) is a rare disease which is characterized by brittle bone, blue sclerae and short stature (Schindeler et al., 2022). Most of the OIs result from mutations in the COL1A1 and COL1A2 genes (Chen et al., 2022). Type I collagen is widely present in the bone, skin and tendon tissue, and nowadays, OI is recognized as a collagen-related disorder (Kaneto et al., 2014; Li et al., 2019).

Perthes disease, also known as Legg-Calve-Perthes disease (LCPD), is characterized by necrosis of the femoral head which results from interruption of blood supply to the femoral head in the pediatric population (Mörlin and Hailer, 2021). Although certain genetic components have been reported as contributing factors, its etiology remains elusive (Rodríguez-Olivas et al., 2022). Mutation in factor V Leiden, polymorphisms in prothrombin, and methylenetetrahydrofolate reductase have been attributed to increased risk of LCPD (Buendía-Pazarán et al., 2022; Yasa et al., 2007).

Here, We present a rare case of Perthes disease in a child with osteogenesis imperfecta (OI) caused by a mutation in the COL1A1 gene (*NM_000088):exon25:c.1726C>T, (p.Gln576X)*. To the best of our knowledge, this is the first ever reported case genetically confirmed OI patient with Perthes disease. The genetic mutation in COL1A1 might be an underlying genetic cause of Perthes disease.

## Case Presentation

**FIGURE 1:**
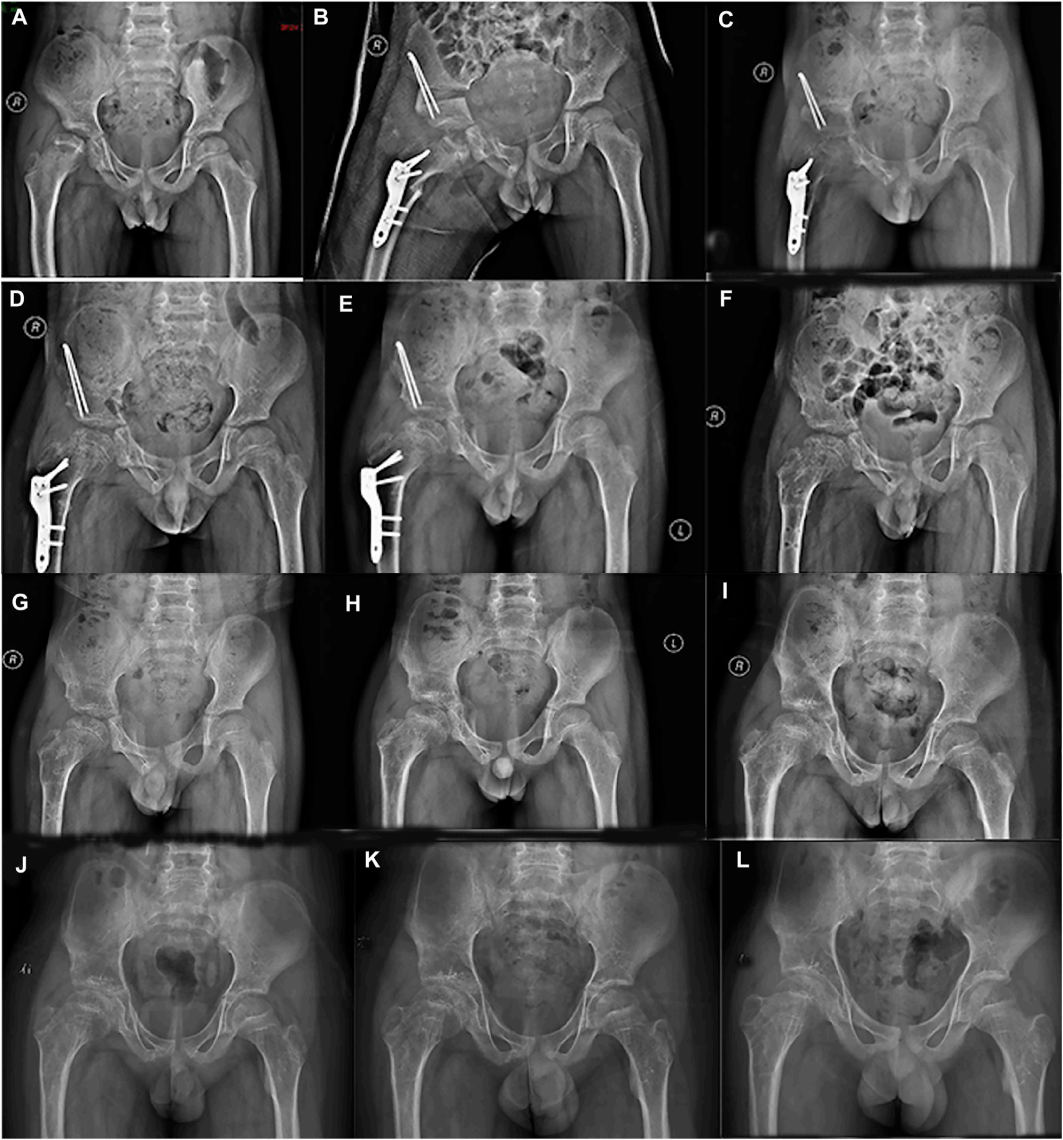
Series radiographs of pelvis from a boy with Perthes disease presentation. **(A)** Pelvic radiograph of a 7.5 years old boy (March 2016). **(B)** Postoperative pelvic radiograph (March 2016). **(C)**. 4 months after operation (July 2016). **(D)**. 7 months after operation (October 2016). **(E)** 11 months after operation (February 2017). **(F)**. 12 months after primary operation, and 1 month after implant removal (March 2017). **(G)** 3 months after implant removal (April 2017). **(H)** 1 year after implant removal (March 2018). **(I)** 2 years after implant removal (January 2019). **(J)** 3 years after implant removal (November 2019). **(K)** 4 years after implant removal (October 2020). **(L)** 5 years after implant removal (January 2022).

**FIGURE 2:**
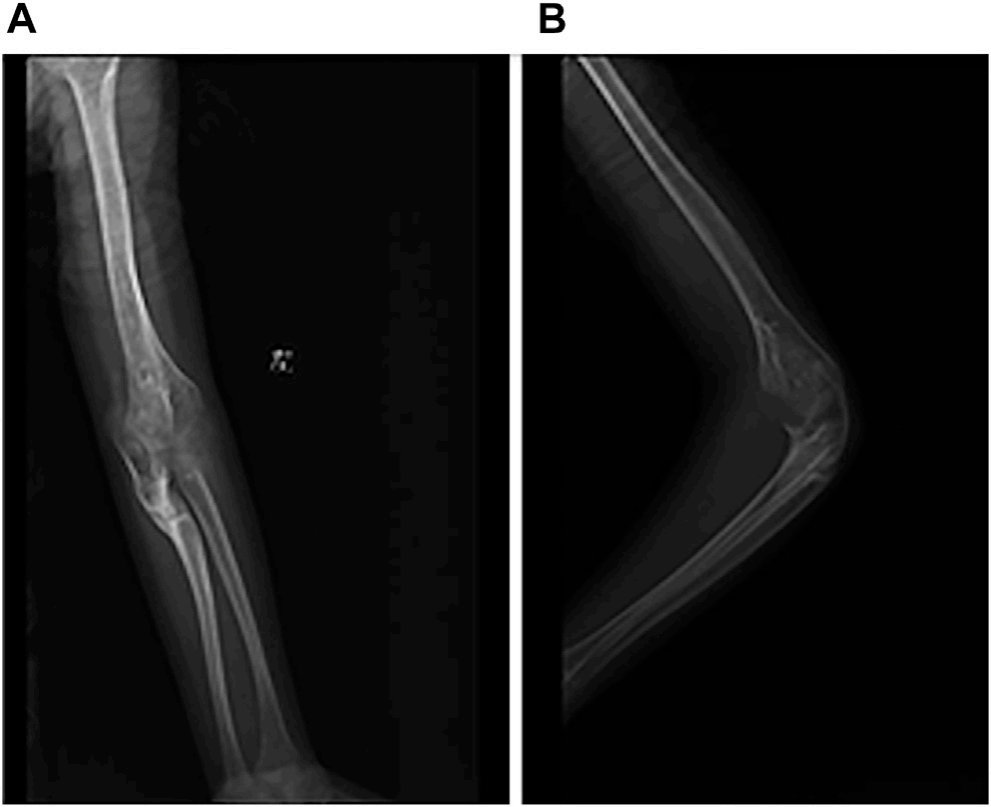
Radiograph of left elbow joint from conservative treatment. **(A)** AP view of elbow joint. **(B)** Latereal view of elbow joint.

A 7-year-old boy was treated in our hospital in March 2016 with a history of chronic pain in the right hip joint and limping for a year. He was radiologically diagnosed as Perthes disease. He underwent right acetabular osteotomy and ipsilateral proximal femoral varus osteotomy in order to improve the femoral head containment in the acetabulum. Series radiographs in the subsequent follow-ups demonstrated the progression of Perthes disease (see Figure 1). In the past few years, he suffered from multiple fractures in his left upper extremity around elbow joint; however, he did not undergo any surgical treatment (see Figure 2). He suffered from right tibial shaft fracture in November 2019, and it was treated with closed reduction and elastic stable intramedullary nails (ESINs) at a local hospital. Unfortunately, he was presented to our hospital with his right femoral shaft fracture in February 2022. Prior to the fracture of his right femur, he had a normal range of motion (ROM) of the hip without limping, and the pelvic X-ray demonstrated Stulberg Type II hip joint with a round femoral head.

Despite having multiple fractures over the period of time, he had neither undergone a complete evaluation in order to diagnose OI nor received any pharmacological intervention for fragility fracture. However, the Z-value on the DEXA (Dual Energy X-ray Absorptiometry) scan in the latest admission was -2.8.

**FIGURE 3:**
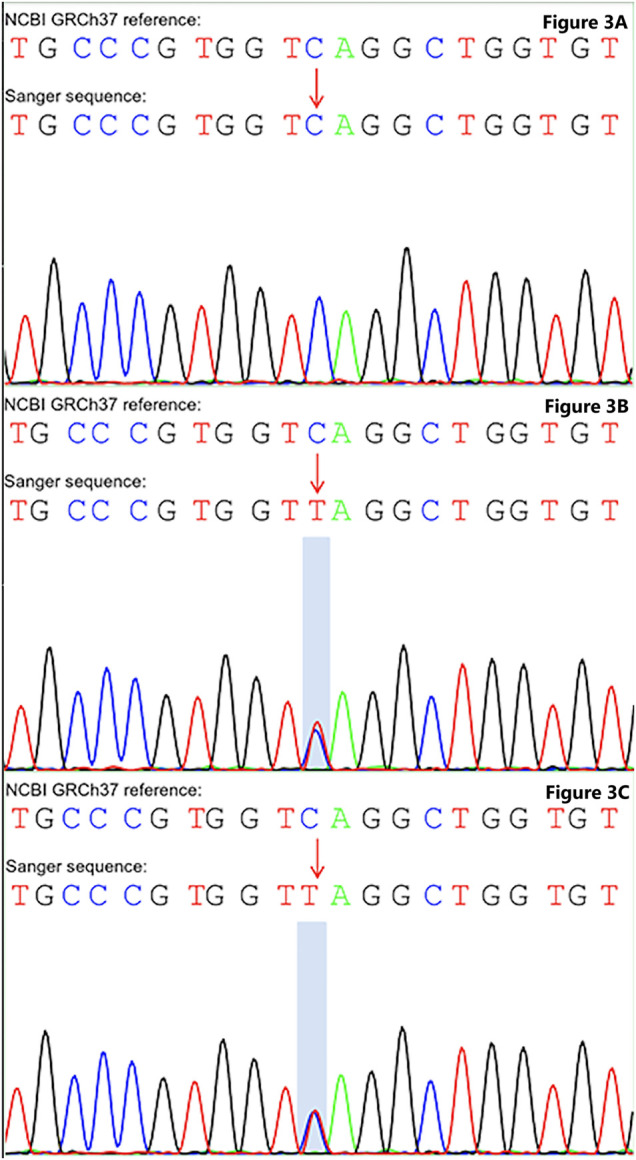
Sanger sequencing unravel gene mutation in COL1A1. **(A)** Wild type was detected in the COL1A1: NM_000088: exon25: c.C1726T: p.Q576X without mutation in the father. **(B)** Heterozygous mutation in the COL1A1: NM_000088: exon25: c.C1726T: p.Q576X of the mother. **(C)** Heterozygous mutation in the COL1A1: NM_000088: exon25: c.C1726T: p.Q576X of the patient.

**FIGURE 4:**
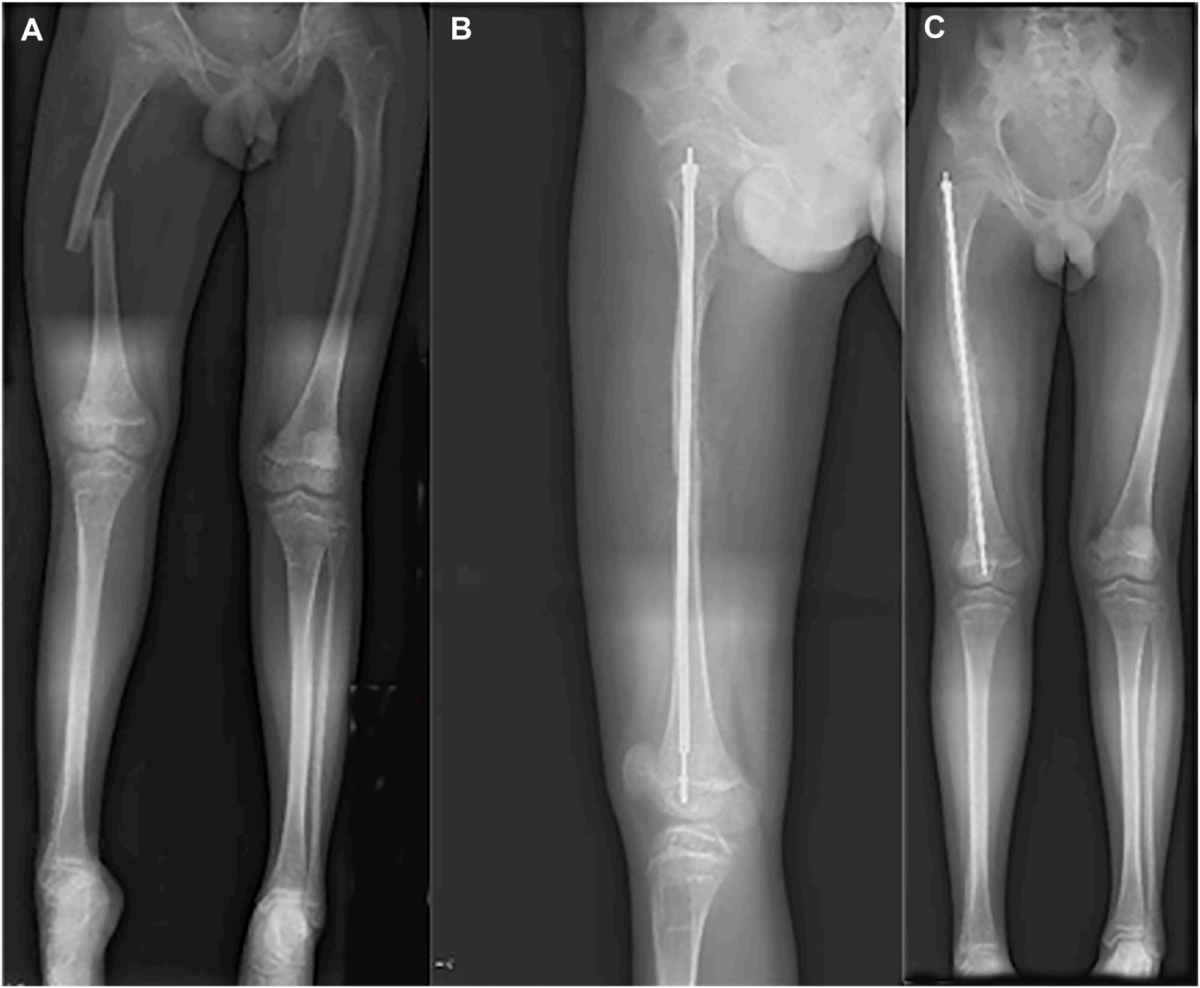
Radiograph of 13-year-old boy with right femoral fracture. **(A)** Full-length AP view of lower extremity before surgery. **(B)** Lateral of femur after surgery. **(C)** Full-length AP view of lower extremity after surgery.

In the latest admission, the patient’s height was 150 cm and body weight was 45 Kg. On inspection, a slightly bluish sclerae were noticed, and an abnormal appearance and limited ROM of the left elbow were evident. After reviewing his medical history, he was suspected of having OI. Subsequently, gene mutation in COL1A1 (OMIM 166200) was unraveled by whole exome sequencing (see Figure 3). Therefore, telescopic nailing was used to treat the femoral shaft fracture (see Figure 4). After the nailing of the right femur, the appearance of the lower extremity seemed normal and symmetrical.

As for the detailed methodology of genetic analysis, we collected 2 ml of whole blood (EDTA anticoagulation) of the children and their parents, and the samples were sequenced by Xiamen Jiyuan medical laboratory. Subsequently, we extracted the whole genomic DNA of peripheral blood leukocytes, built the DNA library, and carried out 150 bp double terminal sequencing. After analyzing the original data of sequencing by biological information, the detected single nucleotide polymorphism and indel variants were annotated by ANNOVAR software.

The annotation information includes the start and end positions of the chromosome, reference alleles, alternative alleles, gene function, population frequency in the public database (including thousand human genome, ExAC, gnomAD database, etc.), protein function prediction (including revert, ClinPred, SIFT, provian, mutation taster, polyphen2, dbscsnv11, etc.). Furthermore, we followed the classification standards and guidelines of genetic variation of the American College of medical genetics and genomics to evaluate the pathogenicity and genetic interpretation of candidate gene variation.

According to the whole exome sequencing analysis, we found the mutation c.1726C>T: p.Gln576X (NM_000088) in exon 25 of the COL1A1 gene. This was a heterozygous nonsense mutation that base 1726 changed from cytosine C to thymine T, resulting in the mutation of amino acid 576 from glutamine to stop the codon from forming the truncated protein. Besides, the detected variation had been reported in the OI & Ehlers-Danlos syndrome variant databases, but the variation has not been reported in the literature before. The upstream and downstream primers were designed for the candidate ectopic sites, and the polymerase chain reaction was carried out, which was verified by Sanger sequencing. Primer design: COL1A1-F, 5′-CTC​CCA​AGA​TGC​CCT​TCC​AG-3’; COL1A1-R, 5′-TCT​CCC​CAA​GTC​CCA​CTC​AT-3’. Sanger sequencing was performed on the patient and their parents, suggesting that the variation was inherited from the mother (see Figure 3). Combined with the results of whole exome sequencing and Sanger sequencing, the mutation was determined as a gene mutation with possible pathogenicity according to American College of medical genetics and genomics guidelines (the evidence level of pathogenicity was pvs1 + pm2_supporting).

## Discussion and Conclusion

We presented a boy with OI caused by a heterozygous mutation in the COL1A1 gene (NM_000088):exon25:c.1726C>T, (p.Gln576X). Series radiographs of the hip joint displayed the characteristics of Perthes disease.

OI is a genetic disorder caused by an abnormal production or structure of collagen type I (Nijhuis et al., 2022). The incidence of this rare condition is 1 in 15,000–20,000 births (Forlino and Marini, 2016). Autosomal dominant, recessive inheritance and X autosomal inheritance patterns have been reported. The severity of symptoms varies widely between different types of OI, ranging from mild symptoms with very few fractures with a normal quality of life to severe symptoms with frequent fractures, severe physical incompetence and decreased life span (Nijhuis et al., 2019; Robinson and Rauch, 2019). The patient had a normal height and relatively normal bone X-ray images in our study. Not only had the boy suffered multiple fractures in the past few years, but his mother, a heterozygous carrier, also experienced one fracture in adolescence and had a mild OI. Therefore, this mutation appeared to be pathogenic, leading to mild type I OI in this pedigree.

Perthes disease has been reported and studied for more than 100 years, but its etiology remains unknown (Pavone et al., 2019). Between 1909 and 1910, LCPD was described almost at the same time by different authors, Arthur Legg, Jacques Calve, Georg Perthes and Henning Waldernstrom (Hailer and Hailer, 2018). Several theories, including environmental, metabolic and genetic predilection, have been proposed as the causative factor for this disease (Leroux et al., 2018; Ibrahim and Little, 2016). Underprivileged social and economic status is associated with the incidence of LCPD in children (Perry et al., 2012a; Perry et al., 2012b), but this is not the scenario for our patient. Obesity has also been identified as a significant risk factor for LCPD (Neal et al., 2016), but this patient presented with average body weight and normal body mass index (BMI).

Presentation of Perthes disease has been reported in certain patients with increased susceptibility (Maleki et al., 2021; Miyamoto et al., 2017). Genetic mutation of COL2A1 has been reported to be involved in the genesis of a type II collagenopathy and a possible cause of LCPD (Kannu et al., 2011; Li et al., 2014). Besides, factor V Leiden has been reported to have an increased risk of Perthes disease, but the result was not significant (Szepesi et al., 2004; Glueck et al., 2007). Moreover, a child with Kienbock’s disease and factor V thrombophilia has been reported to develop LCPD (Baltzer et al., 2016). However, the available literature on substantial heterogeneity reached no unanimity on the etiology of LCPD (Woratanarat et al., 2014; Vosmaer et al., 2010; Balasa et al., 2004; Kenet et al., 2003).

The hip disorder has been reported in patients with OI, including hip dysplasia, acetabular protrusio, and pseudo-protrusio acetabular deformity (Kishta et al., 2017; Ahn et al., 2019; Mandel et al., 2020; Song et al., 2021). However, only one study reported a patient with OI associated with Perthes disease in 2003 (Petra and Benson, 2003). However, the follow-up of that patient did not fully demonstrate the progress of Perthes, and the diagnosis of OI was not genetically confirmed. In contrast, the patient in our study demonstrated classic progression of LCPD, and containment surgery was performed to maintain the femoral head within the acetabulum. In the latest follow-up, the radiograph of the hip joint demonstrated Stulberg type II. Moreover, the genetic test unraveled a mutation in the COL1A1 gene (NM_000088):exon25:c.1726C>T, (p.Gln576X), and confirmed the diagnosis of OI. Therefore, our study demonstrated the mutation leading to the abnormal structure or function of collagen type I, resulting in decreased mechanism strength, which should be further studied and clarified in the pathogenesis of the LCPD.

There are certain limitations in our study. Firstly, we did not perform genetic tests for the entire family members, including the grandparents. Besides, future investigations, including animal models, might be warranted to explore the significance of this genetic mutation. Moreover, Perthes disease might be a comorbidity of OI rather than a secondary disease.

In conclusion, we report an OI child with heterozygous c.1726C>T, (p.Gln576X) mutation in the COL1A1 gene who later had been diagnosed as having Perthes disease. This study revealed that there might be an association between OI and Perthes disease. Our case report enriches the phenotypes of osteogenesis imperfecta and provides insight into the pathogenesis of LCPD.

## Data Availability

The datasets for this article are not publicly available due to concerns regarding participant/patient anonymity. Requests to access the datasets should be directed to the corresponding authors.
